# Synthesis, Antibacterial and Thermal Studies of Cellulose Nanocrystal Stabilized ZnO-Ag Heterostructure Nanoparticles

**DOI:** 10.3390/molecules18066269

**Published:** 2013-05-28

**Authors:** Susan Azizi, Mansor B. Ahmad, Mohd Zobir Hussein, Nor Azowa Ibrahim

**Affiliations:** Department of Chemistry, Faculty of Science, University Putra Malaysia, 43400 UPM Serdang, Selangor, Malaysia; E-Mails: mzobir@science.upm.edu.my (M.Z.H.); norazowa@science.upm.edu.my (N.A.I.)

**Keywords:** cellulose nanocrystals, ZnO-Ag nanoparticles, antibacterial, thermal properties

## Abstract

Synthesis of ZnO-Ag heterostructure nanoparticles was carried out by a precipitation method with cellulose nanocrystals (CNCs) as a stabilizer for antimicrobial and thermal studies. ZnO-Ag nanoparticles were obtained from various weight percentages of added AgNO_3_ relative to Zn precursors for evaluating the best composition with enhanced functional properties. The ZnO-Ag/CNCs samples were characterized systematically by TEM, XRD, UV, TGA and DTG. From the TEM studies we observed that ZnO-Ag heterostructure nanoparticles have spherical shapes with size diameters in a 9–35 nm range. The antibacterial activities of samples were assessed against the bacterial species *Salmonella choleraesuis* and *Staphylococcus aureus*. The CNCs-stabilized ZnO-Ag exhibited greater bactericidal activity compared to cellulose-free ZnO-Ag heterostructure nanoparticles of the same particle size. The incorporation of ZnO-Ag hetreostructure nanoparticles significantly increased the thermal stability of cellulose nanocrystals.

## 1. Introduction

Recently, heterostructure nanoparticles have been considered of broad interest owing to their potential applications in nanodevices [1], biomedicine [2], and photocatalysis [3,4,5]. Among the diverse heterostructure materials, metal-semiconductors are one of the most general heterostructures because of their unique optical, electrical, biomedical, and catalytic properties [6]. Compared to all other metal-semiconductor materials, ZnO-Ag has received considerable attention, not only because ZnO with various nano-sized structures can be simply made via a series of simple processes [7], but also because Ag nanoparticles have good chemical and physical properties. Nano-sized ZnO is a bactericide and inhibits both Gram-positive and Gram-negative bacteria [8]. Furthermore, doped Ag reduces the ionization energy of acceptors in ZnO and consequently enhances the emission [9]. Therefore, Ag ions can enhance the antimicrobial ability of ZnO [10,11]. 

In recent years, the use of nanofibular materials as a scaffold to prepare metallic nanoparticles has merited substantial attention owing to their important potential applications in the fields of catalysis, electronic nanodevices, optoelectronics, sensors, biomedical, and nanocomposites [12,13]. Distinct from conventional stabilizers, the physical size of the nanofabular materials and embedded particles are both in the submicrometer or nanometer range, thus the characteristic large surface area of nanofibular stabilizer and nanoparticles is maintained [14]. Furthermore, these hybrids can possess both the advantages of nanofibers, such as light weight, flexibility and moldability, and of inorganic particles such as exceptional functionality, high strength and thermal stability.

Cellulose nanocrystals, which are typically crystalline rod-like particles, can be easily extracted from a variety of renewable sources by controlled acid hydrolysis of cellulose. They have some notable properties, such as large aspect ratio, good dissolvability in water, excellent mechanical properties, and a high capacity for absorption of guest molecules and cations [15]. Cellulose nanocrystals have found applications in material science, for instance, the reinforcement of polymers [16], however, they have low thermal stability, which limits their applications as reinforcements. The use of inorganic nanoparticles may enhance the thermal properties of CNCs which can be useful for high performance applications. In addition, a small number of biomedical applications have been reported for cellulose nanocrystals. Only one study shows that CNCs can be used for preparing colloidal suspensions with strong antimicrobial power [17]. 

In this study, for the first time, ZnO-Ag heterostructure nanoparticles were synthesized with cellulose nanocrystals as a new stabilizer in order to prevent the formation of aggregated ZnO-Ag hetreostructure nanoparticles and improve the stability of the nanoparticle dispersion. These ZnO-Ag/CNCs materials have been examined for their antibacterial and thermal properties. It was expected that the synthesized ZnO-Ag/CNCs hetreostructure nanoparticles would exhibit excellent antimicrobial, thermal, and mechanical properties, and then, the synthesized ZnO-Ag/CNCs materials might be used in the fields of biomedicine and nanocomposites as a good antimicrobial and multifunctional filler, respectively.

## 2. Results and Discussion

The sequential addition of zinc acetate dihydrate (Zn(AcO)_2_·2H_2_O) and AgNO_3_ is important for the formation of Ag nanoparticles on ZnO surface [18]. CNCs have good dispersability in water and the suspension does not sediment owing to the plentiful hydroxyl groups and as a consequence of the sulfate groups on the surface of CNCs introduced during the sulfuric acid hydrolysis [19]. The first stage involves absorption of Zn^2+^ cations onto the negatively charged hydroxyl (OH) functional groups, through electrostatic interactions between oxygen atoms of the polar hydroxyls and metallic cations. These effects control the size by inhibiting the agglomeration of metallic particles formed in the synthesis process. Consequently nanoparticles which are synthesized in CNCs may be ideally small. With the drop wise addition of NaOH, Zn(OH)_2_ is slowly formed. Under thermal conditions, ZnO is formed. With the addition of AgNO_3_, Ag^+^ ions are reduced to Ag nanoparticles in alkaline suspension. The possible reactions are as follows:


Zn^2+^---CNCs + 2OH^-^ → Zn(OH)_2_---CNCs (1)

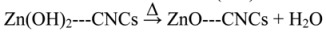
(2)


2CNCs....ZnO + 2Ag^+^ + 2(OH)^-^ → 2CNCs....ZnO....Ag  + H_2_O + 1/2O_2_ (3)

### 2.1. Structure Characterization

**Figure 1:**
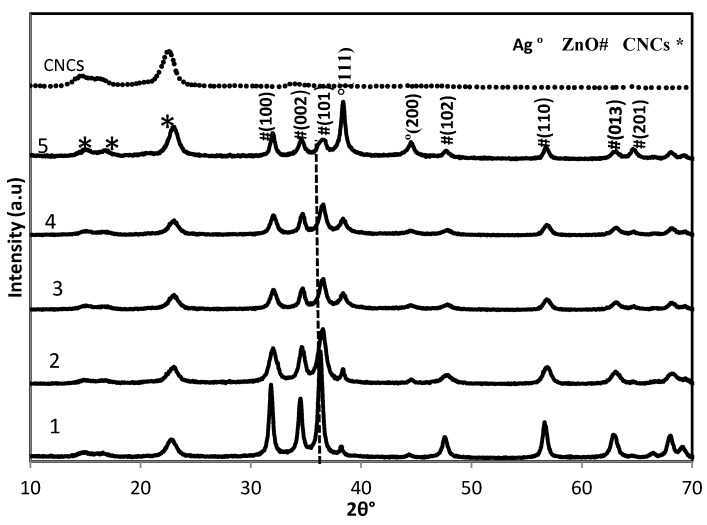
XRD patterns of ZnO-Ag/CNCs and CNCs.

The XRD patterns of original CNCs and CNCs-stabilized ZnO-Ag nanoparticles are shown in Figure 1. Three sets of diffraction peaks corresponding to CNCs, ZnO and Ag are detected in the all ZnO-Ag/CNCs samples. 

The peaks of ZnO and Ag can be assigned to hexagonal wurtzite ZnO and face-centered-cubic (fcc) Ag, which are in agreement with the data of JCPDS 36-1451 and JCPDS 4-0783, respectively. No impurity diffraction peaks are seen in the patterns. Furthermore, there are no important changes in any of the peaks, indicating no ZnO_1-x_Ag_x_O solid solution and collapse of crystal structure in the samples. The slight displacement of ZnO diffraction peaks to higher angles, may be due to the increased defects in the ZnO-Ag interface [20]. This suggests that the obtained nanoparticles are ZnO-Ag heterostructures. In addition, the diffraction peaks of ZnO became gradually broad and weak as the content of Ag is increased, implying the average particle size of the ZnO decreased. In order to calculate the average particle size of the ZnO nanoparticles, we adopt the Scherrer formula using the full width at half maximum (FWHM) value of the ZnO diffraction peaks [21]:


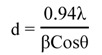
(1)

where d, λ, θ, and β are the average particle size, the XRD wavelength of 0.154 Å, Bragg diffraction angle, and the FWHM of the diffraction peak of ZnO (1 0 1) plane at 36.25°, respectively. The average particle sizes of ZnO nanoparticles are about 28.08, 19.45, 15.86, 12.21, and 5.24 nm for samples 1 to 5, respectively. These results suggest that the effect of different Ag content on the average particle size of ZnO nanoparticles is relatively significant.

**Figure 2:**
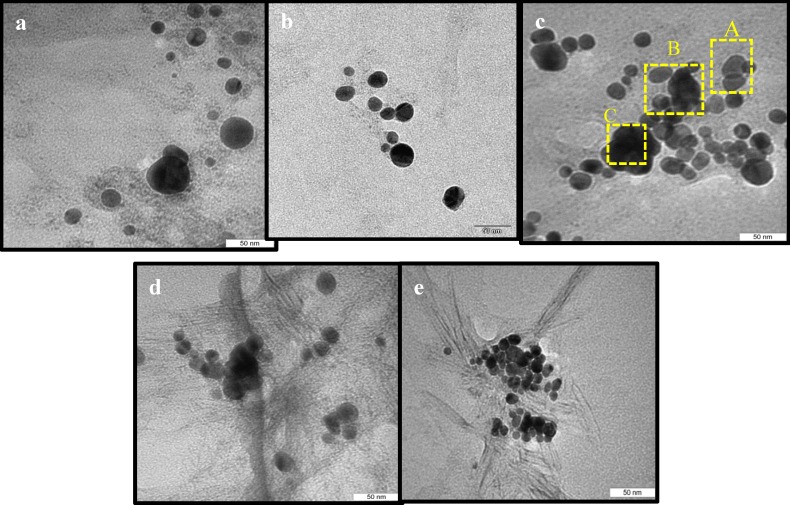
TEM images of ZnO-Ag/CNCs of (**a**) 1.0. (**b**) 3.0. (**c**)5.0. (**d**) 7.0. (**e**) 10.0. wt% of Ag concentrations.

**Figure 3:**
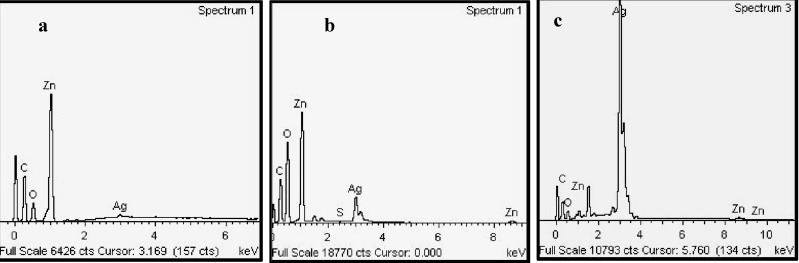
EDS spectra of 5.0 wt% ZnO-Ag nanoparticles at circles marked (**a**) A. (**b**) B. and (**c**) C from Figure 2c.

The nanostructure of the samples was characterized by using transmission electron microscopy (TEM). The TEM images shown in Figure 2a–e are for ZnO-Ag heterostructures stabilized in CNCs with different silver loading contents, in which some transparent and dark particles were found. It can be seen that, for each sample, there are small sized spherical particles with a narrow size distribution relatively well dispersed within the CNCs. This is in agreement with the effect of CNCs for controlling the size and preventing aggregation of metallic nanoparticles. The detailed composition of particles was characterized by using EDS analysis (Figure 3a–c). From the EDS spectrum (Figure 3a) it was found that the signals of C, Zn, and O, but not Ag, were observed in particles with transparent areas (Figure 2c, zone A), indicating these particles are related to ZnO. At the interface, between dark and transparent areas (Figure 2c, zone B), the peaks of C, Zn, O, and Ag were found (Figure 3b).

The EDS spectrum (Figure 3c) from the dark area (Figure 2c, zone C), indicates the peak of Ag is strong while the signals of Zn and O are barely observable. These results show that the silver nanoparticles with dark shades are attached on the surface of transparent ZnO particles, and, free Ag nanoparticles are barely observed, indicating the successful combination of Ag nanoparticles with ZnO particles. The TEM images show that the size of particles decreased with increased Ag content. The average particles sizes are about 34.76, 26.20, 21.72, 17.46 and 8.83 nm for samples 1, 2, 3, 4, and 5, respectively. Therefore, Ag may have an inhibitory effect on the growth of zinc oxide crystallites. These results are in agreement with the obtained average particle size of ZnO nanoparticles from XRD patterns. This inhibition effect of silver particles also was reported by Lin and coworkers [22] when Ag nanoparticles were doped on the surface of cuprous oxide crystallites.

**Figure 4:**
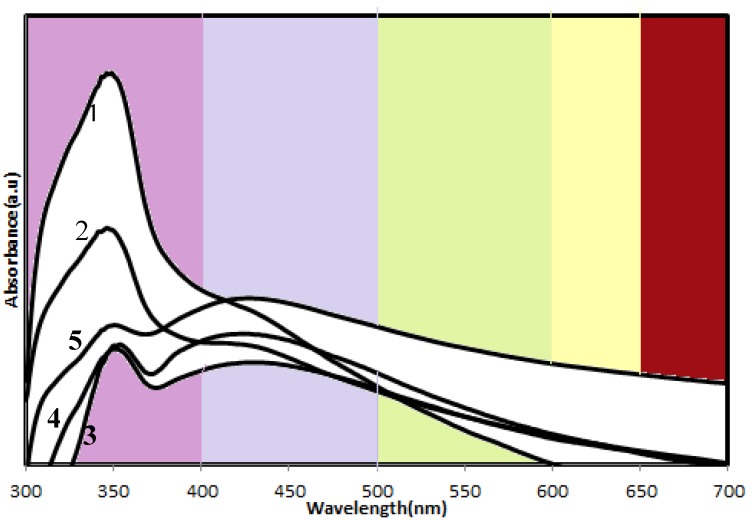
UV-vis absorbance spectra of ZnO-Ag/CNCs samples.

The optical properties of the samples were measured by UV–vis absorption spectroscopy. From Figure 4 is observed that the ZnO-Ag nanoparticles show two absorption peaks in the UV and visible regions. The absorption at visible wavelengths increases with increasing Ag content because of the surface plasmon resonance of the Ag nanoparticles, confirming the presence of Ag nanoparticles which is consistent with the XRD and TEM results. On the other hand, the range and intensity of the absorption in the UV wavelength, which is assigned to the absorption of ZnO crystals, decreases with increasing Ag content due to the reduced absorption of ZnO particles in the presence of a thick layer of Ag nanoparticles. A similar observation also was reported by Yin and coworkers [23] for Ag-ZnO nanocomposites. The absorption results were also in agreement with the visual inspection—the color of the powder shifts from light purple to dark red, with increasing Ag loadings from sample 1 to 5. 

### 2.2. Thermogravimetric Analysis

**Figure 5:**
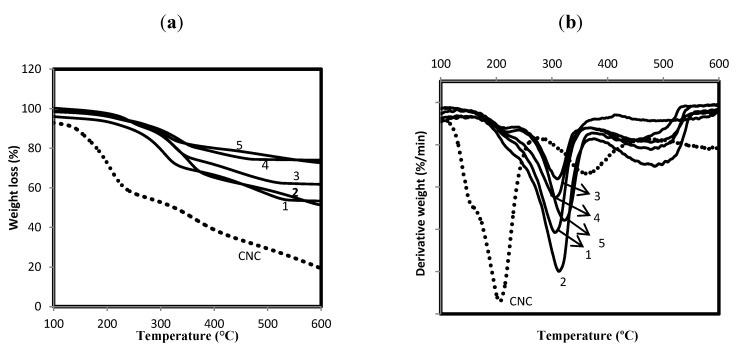
(**a**) TGA and (**b**) DTG thermograms of ZnO-Ag/CNCs and CNCs.

Thermogravimetric analysis was performed in order to determine the effect of the synthesized ZnO-Ag heterostructure nanoparticles on the thermal stability of the cellulose nanocrystals. Figure 5 shows the TGA and DTG curves of the original cellulose nanocrystals and the ZnO-Ag/CNCs samples. 

The thermal decomposition pathways of the cellulose nanocrystals include depolymerisation, dehydration, and decomposition of glycosyl units, followed by the formation of a charred residue [24]. The two main steps observed in the ZnO-Ag/CNCs nanoparticles correspond to the thermal degradation of the CNCs [25], which started around 138 °C with a maximum rate of weight loss at 201 °C and 20.43 wt% residue at the end of the degradation. The onsets of thermal degradation for the ZnO-Ag/CNCs were found at higher temperatures, with maximum rates of weight loss at 300, 304, 312, 314 and 320 °C with 56.29, 61.33, 63.48, 75.90, and 78.59 wt% residues at the end of degradation for the samples 1, 2, 3, 4, and 5, respectively. It can be seen that thermal stability of CNCs was increased by about 100 °C with the incorporation of ZnO-Ag nanoparticles, and was more considerable for the smaller sized ZnO-Ag nanoparticles with greater surface area. These improvements can be assigned to the high interaction between cellulose nanocrystals and ZnO-Ag nanoparticles. The ZnO-Ag nanoparticles with high aspect ratio are able to preserve the temperature and inhibit the heat transmission efficiently. The amount of ZnO-Ag nanoparticles was estimated by comparing the percentage residue of the ZnO-Ag/CNCs samples and the CNCs at the end of degradation. The content of ZnO-Ag was estimated to be about 15.43, 20.47, 22.62, 35.04, and 37.73 wt% for samples 1, 2, 3, 4, and 5, respectively.

### 2.3. Antibacterial Assessment

**Figure 6:**
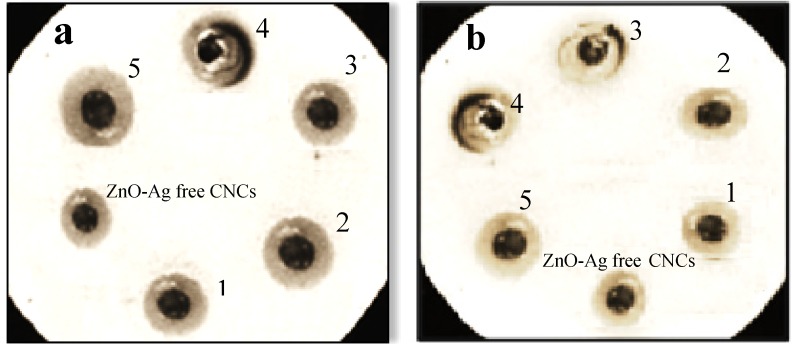
Inhibition zone of ZnO-Ag/CNCs and ZnO-Ag free-cellulose against (**a**) Gram-positive and (**b**)Gram-negative bacteria.

The antibacterial ability of the ZnO-Ag/CNCs samples was determined in terms of the inhibition zone created on agar around the paper discs as shown in Figure 6a,b. Table 1 shows the average diameters of the inhibition zones of all ZnO-Ag/CNCs samples and ZnO-Ag heterostructure nanoparticle-free cellulose against *Staphylococcus aureus* and *Salmonella choleraesuis*. As shown in Table 1, with the increase of Ag content, the diameter (D) of the inhibition zone increased gradually. The small crystal size and large surface area of the heterostructure nanoparticles may contribute to antibacterial enhancement. The antibacterial ability of ZnO-Ag is related to the photocatalysis and metal release process [26,27]. When ZnO nanoparticles are under light irradiation, electron–hole pairs are created. The hole (h^+^) reacted with OH^−^ on the surface of nanoparticles, generating hydroxyl radicals (OH^•^), superoxide anion (O^2−^) and perhydroxyl radicals (HO_2_**^•^**). These highly active free radicals harmed the bacterial cells resulting in decomposition and complete damage [28]. It can be seen that the antibacterial ability of the ZnO-Ag/CNCs samples was stronger against the Gram-positive *Staphylococcus aureus* compared to the Gram-negative *Salmonella choleraesuis*. Stronger antibacterial power against Gram-positive bacteria has also been formerly reported [29,30,31]. The cell walls of Gram-negative bacteria contain an external lipopolysaccharide (LPS) membrane that shields the peptidoglycan layer. Furthermore, it helps bacteria to survive in environs where exterior materials exist that can damage them. Furthermore, ZnO-Ag heterostructure nanoparticles (35 nm) without CNCs displayed a less powerful influence toward Gram-positive and negative bacteria in comparison to sample 1. Our outcomes suggest that the activity of ZnO-Ag/CNCs could be the result of a high contact of the well dispersed and stabilized heterostructure nanoparticles with the bacteria, through a tight joining of CNCs to the bacterial envelope.

**Table 1:** Inhibition zone of ZnO-Ag/CNCs and ZnO-Ag free-cellulose.

Sample	Diameter of zone (mm)	
	Gram-positive Staphylococcus aureus	Gram-negative Salmonella choleraesuis
1	10.8	9.4
2	11.4	10.3
3	11.3	10.5
4	13.5	11.8
5	13.6	12.7
ZnO-Ag	9.1	8.7

## 3. Experimental

### 3.1. Materials

All the chemicals were analytical grade and used as received without more purification. Cotton cellulose from filter paper (Q1, Whatman) was supplied by Fisher Scientific (Pittsburgh, PA, USA). Sulfuric acid (95%–98%, reagent grade) was purchased from Scharlau (Barcelona, Spain). Ethanol and sodium hydroxide were provided by Sigma Aldrich (St. Louis. USA). Zinc acetate dehydrate (99%) and silver nitrate (99.98%), used as precursors, were provided by Merck (Darmstadt, Germany). All the solutions were prepared with deionized water.

### 3.2. Extraction of Cellulose Nanocrystals

Extraction of the CNCs was carried out according to a previous work [32]. The cellulose powder harvested from one filter paper (2 g) was hydrolyzed with a sulfuric acid solution (20 mL, 64 w/w%) at 45 °C for 60 min. The resultant suspension was diluted 10-fold with cold water (4 °C) followed by centrifugation and dialysis until a neutral pH was reached. Finally, the sample was freeze-dried.

### 3.3. Preparation of ZnO-Ag/CNCs Nanoparticles

The ZnO-Ag/CNCs was synthesized as follows: Zn(AcO)_2_·2H_2_O alcoholic solution samples (50.0 mL, 10.0 wt%) were dispersed to five separated CNCs suspensions (100.0 mL, 2.0 wt%) by magnetic stirring. After complete mixing, a sodium hydroxide solution (5.0 mol/L) was added dropwise to the mixed solutions under continuous stirring at 80 °C until pH > 10 was reached. After observing a milky color suspension, aqueous AgNO_3_ solution samples with different concentrations (20 mL, 1.0, 3.0, 5.0, 7.0, 10.0 wt%, relative to Zn(AcO)_2_·2H_2_O), were separately to the five above suspensions (sample 1, 2, 3, 4, and 5, respectively) under strongly stirring, and the reaction was continued for 2 h. The products were collected through centrifugation and careful washing three times with distilled water. The final products were obtained by drying at 100 °C for 1 h for complete transformation of the remaining zinc hydroxide to zinc oxide.

### 3.4. Antibacterial Activity Testing

The samples were assessed for antibacterial ability against the Gram-negative sp. *Salmonella choleraesuis* and the Gram-positive one *Staphylococcus aureus*. Six paper discs, containing 5 μL of the ZnO-Ag/CNC suspensions were placed onto an agar plate that was inoculated with bacteria. Ampicillin and streptomycin were used as standard antibacterial agents for negative and positive inhibitory controls, respectively. The bacterial inoculum was standardized to 0.5 MF units, which meant that approximately 10^8^ colony-forming units of each bacterium were inoculated on a plate. The plates were inverted and incubated under light at 37 °C for 24 h, then the zone of complete inhibition was measured to the nearest whole millimeter, using sliding calipers or a ruler held on the back of the inverted petri plate. Three replicate tests were carried out in the same conditions for each sample. Furthermore, an assay for cellulose-free ZnO-Ag heterostructure nanoparticles with particle size of 35 nm was also carried out to show the advantage of using CNCs on the antimicrobial power of ZnO-Ag nanoparticles.

### 3.5. Characterization

Wide-angle X-ray diffraction (WXRD) patterns of the ZnO-Ag/CNCs and the CNCs were recorded using an XPERT-PRO diffractometer at 40 kV and 30 mA from 10° to 80° with nickel-filtered Cu (λ = 1.542 Å) at room temperature. The size and morphology of the ZnO-Ag/CNCs were seen using a Hitachi H-700 transmission electron microscope with an acceleration voltage of 120 kV at room temperature. The TEM sample was prepared via dropping the sample suspension on a Cu grid coated with carbon film, and then the specimens were negatively stained with 1% uranyl acetate and allowed to dry at room temperature. The components of samples were evaluated by the energy-dispersive X-ray spectroscopy (EDS). The UV-visible spectra of the nanoparticles were recorded over the range of 200 to 800 nm with a Lambda 25-Perkin Elmer UV-vis spectrophotometer. The thermal behavior of the ZnO-Ag/CNCs powders was recorded with a thermogravimetric analyzer TGA7 (Perkin-Elmer) in a nitrogen atmosphere at a heating rate of 10 °C/min from 25 to 600 °C.

## 4. Conclusions

ZnO-Ag heterostructure nanoparticles with a spherical shape and average size of less than 35 nm were successfully synthesized in cellulose nanocrystals (CNCs). The antibacterial tests showed that CNCs can strongly enhance the antibacterial power of ZnO-Ag heterostructure nanoparticles. The antibacterial results compared favourably with most of other assays conducted with the same species. Furthermore, we demonstrated that the incorporation of ZnO-Ag in CNCs can significantly improve the poor thermal property of CNCs. The synthesized ZnO-Ag/CNCs heterostructure nanoparticles with strong antimicrobial ability and high thermal stability are expected to find notable applications in the pharmaceutical and nanocomposite fields.
